# The University and Its Medical Department

**Published:** 1869-02

**Authors:** 


### EDITORIAL.

### THE UNIVERSITY AND ITS MEDICAL DEPARTMENT.

The subject of a Medical Department in connection with the Literary Department at Iowa City, is creating considerable discussion at this time. We had written an article for this number of the *Journal*, but for the want of room are compelled to withhold it until the next issue. Should our Literary and Law Departments at Iowa City be so unfortunate as to have their quiet disturbed by a Medical Department, we trust that it will fare better in the Association than the Medical Department of the Michigan University, connected with the Literary and Law Departments, at Ann Arbor. The Medical and Surgical Reporter, of Philadelphia, Dec. 19th, speaks as follows:

"The State authorities by their course toward the Medical Department of the University of Michigan, are keeping that University in such a constant turmoil and excitement, that some of the profession are disgusted with it, and it looks as if the regular profession might yet withdraw from all connection with it. They certainly will, if the policy is pursued of forcing irregular practitioners into position in the institution. This unsettled and uneasy feeling in connection with the Medical teaching in that State, has probably led to the organization of the Detroit School of Medicine," etc.

When Medical Schools are wholly under State patronage, and associated with the Literary and Law Departments, as exist in Michigan, and which the trustees of the University now propose to adopt in connection with our University, it cannot but result in disaster to the School and the best interests of the profession.

Unless our Trustees will provide for all the *pathies* and *isms* in their organization, the friends of those severals *isms* will be on the alert when appropriations are asked, for the support of this department. It is not to be supposed that the several Legislative Assemblies which shall hereafter convene in our State, will be a unit in thought, word and action on the subject of Medical Education. I remember very well, at a meeting of our State Legislature in Iowa City, (the old capitol,) we succeeded in securing the passage of a bill appropriating to the College of Physicians and Surgeons at Keokuk, known as the Medical Department of the Iowa University, the sum of $5,000. But imagine our surprise when the bill was vetoed by the Governor, and in his message accompanying the veto, he gave as one of his principal reasons for the course pursued that the Legislature had no right to favor by their appropriations from the State funds one class of medical men over that of another. The same argument will be used, should the Trustees of our University carry out the plan proposed, and prejudice not only the Medical Department, but the interests of the other departments associated with it.

